# Vincristine-Induced Peripheral Neuropathy in Children With Malignancy and the Effect of Missed Doses on Treatment Success

**DOI:** 10.7759/cureus.46063

**Published:** 2023-09-27

**Authors:** Nurşah Eker, Gulten Ozturk

**Affiliations:** 1 Pediatric Hematology and Oncology, Marmara University Medical Faculty, Istanbul, TUR; 2 Pediatric Neurology, Marmara University Medical Faculty, Istanbul, TUR

**Keywords:** chemotherapy, children, cancer, peripheral neuropathy, vincristine

## Abstract

Introduction: Recognizing the symptoms of vincristine-induced peripheral neuropathy (VIPN) earlier is crucial to preventing the persistent neurological sequelae. The treatment of neuropathy is to discontinue the drug, and the effect of a missed dose of vincristine on treatment success is unclear. This study aims to evaluate VIPN in children with malignancy and the effect of skipping vincristine doses on the treatment success of patients at a single center, retrospectively.

Methods: Medical records of the children with cancer who received vincristine in our institution between 2013 and 2020 were analyzed retrospectively.

Results: Vincristine neuropathy was found in 42 (7%) of 598 pediatric patients who received at least one dose of vincristine during the study period. Neuropathy developed at a statistically significantly lower cumulative dose in patients younger than seven years of age (p=0.04). The mean neuropathy duration of the cases was 8.5 months, and the findings of 40 (95.2%) cases improved. The mean cumulative dose was higher in patients with diffuse nerve involvement. The missed dose of vincristine was lower in the cases in complete remission compared to the other cases and higher doses of vincristine were missed in the stable disease group than in the remission group (p=0.03).

Conclusion: VIPN can be encountered in less cumulative doses, mainly in the younger age group. Missed doses of vincristine may affect treatment success, and more comprehensive studies are needed to show this effect more clearly.

## Introduction

Survival rates of children with malignancy increase due to new multi-model treatment strategies. Increasing survival rates make the long-term side effects more crucial. One of these side effects is chemotherapy-induced peripheral neuropathy, which could affect 78% of pediatric patients who receive neurotoxic chemotherapy [1]. This neurotoxicity can affect the sensory, motor, and autonomic nervous systems, causing long-term dysfunction and affecting the quality of life. Detecting neurotoxicity earlier is necessary to minimize the severe effects, and taking precautions is very important.

Vincristine is a critical chemotherapeutic drug used frequently in childhood malignancies. It is a vinca alkaloid that may damage the peripheral nerves with acute and long-term side effects [2]. Vinca alkaloids can cause neurotoxicity in 52% of patients [1,3]. Types of vincristine-induced peripheral neuropathy (VIPN) are moderate-to-severe sensory, motor, and autonomic [2]. It is commonly a peripheral sensorimotor neuropathy and is related to the cumulative dose of vincristine [4]. VIPN may be reversible after cessation of vincristine treatment, but it may also persist after treatment [5,6]. Some studies described permanent neurological sequelae like handwriting difficulties and lasting loss of deep tendon reflexes [6]. Recognizing the symptoms of VIPN earlier is crucial to preventing these persistent neurological sequelae.

Although there is no effective treatment for VIPN, the most effective strategy is to discontinue the drug when toxicity is noticed. The effect of discontinuation of vincristine, which is an important agent in pediatric cancer treatment, on treatment success is unclear.

We noticed an increased incidence of VIPN in our patients recently. Therefore, we aimed to evaluate VIPN in children with solid tumors or hematological malignancy and the effect of missed vincristine doses on patients' treatment success at a single center with this study.

## Materials and methods

Medical records of children diagnosed between the ages of 1 and 18 years with cancer and received vincristine according to their chemotherapy protocols in our institution between 2013 and 2020 were analyzed retrospectively. Their medical records were reviewed to collect the following data: age, gender, diagnosis, history of neuropathy before initial diagnosis, symptoms, clinical and nerve conduction study findings, and grade of neuropathy according to the National Cancer Institute Common Terminology Criteria for Adverse Events (NCI CTCAE) [7], cumulative dose of vincristine at the diagnosis of neuropathy. According to NCI CTCAE, grade 1 is asymptomatic, grade 2 is moderate symptoms that limit instrumental activating of daily living (ADL), grade 3 is severe symptoms that limit self-care ADL, assistive device indicated, grade 4 is life-threatening consequences, and grade 5 is death. Patients with neuropathy or neurological sequelae for any other reason before diagnosis with cancer were excluded from the study. The same pediatric neurologist retrospectively re-evaluated the nerve conduction study findings of the patients. Two sensory nerves from the upper extremity and one sensory nerve from the lower extremity were evaluated. A minimum of two motor nerves from the upper extremity and two motor nerves from the lower extremity were studied in all the patients. A standard protocol for nerve conduction study was not present because of the retrospective nature of the study. Diagnosis of neuropathy was made based on clinical and nerve conduction study findings. The treatment and approach for the neuropathy, dose modifications, time to recovery, and the presence of neurological sequela after vincristine-induced neuropathy were also evaluated. In patients, the recurrence of neuropathy was assessed if vincristine was applied again after recovery.

The cases were grouped according to their diagnosis, and the ratio of missed vincristine doses to cumulative vincristine doses according to chemotherapy protocols was examined. While acute leukemia and lymphomas were divided into the hematological malignancy group, other cancers formed the solid tumors group. When evaluating the current status of primary diseases, for solid tumors, cases with no signs of disease as a result of clinical and radiological evaluation were considered as complete remission, cases with progression in existing findings or new findings were regarded as a progressive disease, and cases in which no regression and no progression of findings were detected were considered as stable disease. Bone marrow aspiration and cytogenetic examination results were also evaluated for hematological malignancies, and cases with no evidence of disease and negative cytogenetic results were accepted as complete remission. Conditions that recurred after the complete remission with chemotherapy were grouped as relapsed disease. According to these definitions, the final status of the primary disease of the cases and the effect of missed vincristine doses on treatment success were analyzed. Patients whose parents refused to give consent were excluded from the study. The ethics committee of Marmara University approved the study (2/7/2021, number: 09.2021.894).

Statistical analysis

Data collected was analyzed using SPSS Statistics version 21.0 (IBM Corp. Released 2012. IBM SPSS Statistics for Windows, Version 21.0. Armonk, NY: IBM Corp.). Frequencies (counts) and percentages for nominal variables were presented. The normal distribution of numeric variables was assessed by Kolmogorov-Smirnov/Shapiro-Wilk's tests. Medians and minimum-maximum values were used for the non-normally distributed variables. The Mann-Whitney U test was used to compare differences between two independent groups, and the Kruskal-Wallis test was conducted when three or more independent groups existed. The Mann-Whitney U test was also performed to test the significance of pairwise differences using Bonferroni correction to adjust multiple comparisons. To evaluate the relationship between two categorical variables, Fisher's exact test or Chi-square test, where appropriate, was used. For the investigation of the associations between non-normally distributed variables (cumulative dosage and neuropathy duration), the correlation coefficients and their significance were calculated using the Spearman test. The alpha error was set at 0.05.

## Results

Vincristine neuropathy was found in 42 (7%) of 598 pediatric patients treated with vincristine. The mean age of the cases was 7.73 ± 4.94 years. Considering the relationship between cancer type and polyneuropathy, 21 patients had solid tumors, and 21 had hematological malignancies. These patients received vincristine treatment in accordance with the chemotherapy protocols. The patients' characteristics are shown in Table 1.

**Table 1:** Characteristics of the patients with VIPN (n=42)

	n (%)
Female	16 (38.1%)
Male	26 (61.9%)
Diagnosis	
Medulloblastoma	6 (14.2%)
Optic glioma	9 (21.4%)
Pilositic astrositoma	1 (2.3%)
Oligodenrioglioma	1 (2.3%)
Neuroblastoma	3 (7.1%)
Rhabdomyosarcoma	1 (2.3%)
Acute leukemia	19 (45.2%)
Non-Hodgkin lymphoma	2 (4.7%)
Complaints of the patients	
Gait disturbance	34 (80.9%)
Drooping eyelid	3 (7.1%)
Numbness in the hands	2 (4.7%)
Numbness in feet	1 (2.3%)
Loss of sensation in the legs	1 (2.3%)
Inability to urinate	1 (2.3%)
Finding on physical examination	
Drop foot	36 (85.7%)
Ptosis	3 (7.1%)
Globe vésical	1 (2.3%)
None	2 (4.7%)

It was determined that 2.38% (n=1) had grade 1, 42.8% (n=18) had grade 2, 52.38% (n=22) had grade 3, and 2.38% (n=1) had grade 4 neuropathy according to NCI CTCAE. The median cumulative dose of vincristine received by all patients developing VIPN was 17.23 mg/m². While there was no statistically significant difference between the cumulative dose causing neuropathy and gender (p=0.135), it was found that neuropathy developed at a statistically significantly lower cumulative dose in patients younger than seven years of age, the median age in the study (p=0.04).

The mean neuropathy duration of the cases was 8.5 months, and no statistically significant difference was found when gender and age were compared with the duration of neuropathy (p=0.163), (p=0.644). It was observed that when neuropathy was detected, vincristine was discontinued, pyridoxine was started at 150 mg/m², and the treatment continued until clinical improvement was achieved for all patients. No dose reduction was applied in any of the cases. The findings of 40 (95.2%) cases improved with this application without any sequelae. Neuropathy did not recur when vincristine was started again in any patient whose neuropathy had improved. The other two cases with continued neuropathy findings were lost due to progressive disease.

A nerve conduction study was performed on all patients with neuropathic complaints. Table 2 demonstrates the summary of electrophysiological data of the study group. A needle electromyography study was performed only on some of the patients, but the data was not included as the study was not performed by the same performer, and some of the results were inconsistent because the patients were unwilling to cooperate. The electrophysiological findings of the patients have shown that the type of polyneuropathy detected in all patients was of the axonal type, and predominantly the lower extremities were involved. Pure motor involvement was observed in 88% (n=37) of all patients, pure sensory involvement in 2.4% (n=1), and mixed-type involvement in 9.5% (n=4). Mixed type with sensory and motor polyneuropathy was observed more frequently in hematological malignancies (75% of mixed type is hematological malignancy). The mean cumulative dose was higher in patients with diffuse nerve involvement, but this difference was not statistically significant (p=0.939).

**Table 2:** Electrophysiological findings of the patients SNAP: sensory nerve action potential, CMAP: compound muscle action potential, UE: upper extremity, LE: lower extremity

Patient number	Electrophysiological findings										
	Sensory nerve				Motor nerve						Interpretation
	SNAP*		Velocity		CMAP*		Velocity		F-wave index		
	UE*	LE*	UE	LE	UE	LE	UE	LE	UE	LE	
1	N	N	N	N	low	low	low	low	N	N	Motor axonal
2	N	N	N	N	low	low	N	N	N	N	Motor axonal
3	N	N	N	N	N	low	N	N	N	N	Motor axonal
4	N	N	N	N	N	low	N	N	N	N	Motor axonal
5	N	N	N	N	low	low	N	N	N	N	Motor axonal
6	N	N	N	N	N	low	N	N	N	N	Motor axonal
7	N	N	N	N	N	low	N	N	N	N	Motor axonal
8	N	N	N	N	N	low	N	N	N	N	Motor axonal
9	N	N	N	N	N	low	N	N	N	N	Motor axonal
10	N	N	N	N	low	low	N	N	N	N	Motor axonal
11	N	N	N	N	N	low	N	low	N	N	Mixed
12	low	low	low	low	N	low	N	N	N	N	Mixed
13	N	N	N	N	N	low	N	N	N	N	Motor axonal
14	N	N	N	N	low	low	N	N	N	N	Motor axonal
15	N	N	N	N	low	low	N	N	N	N	Motor axonal
16	N	N	N	N	low	low	N	N	N	N	Motor axonal
17	N	N	N	N	N	low	N	N	N	N	Motor axonal
18	N	N	N	N	N	low	N	N	N	N	Motor axonal
19	N	N	N	N	N	low	N	N	N	N	Motor axonal
20	N	N	N	N	low	low	N	N	N	N	Motor axonal
21	N	low	N	N	N	low	N	N	N	N	Mixedaxonal
22	N	N	N	N	N	low	N	N	N	N	Motor axonal
23	N	N	N	N	N	low	N	N	N	N	Motor axonal
24	N	N	N	N	low	low	N	N	N	N	Motor axonal
25	N	N	N	N	N	low	N	N	N	N	Motor axonal
26	N	N	N	N	low	low	N	N	N	N	Motor axonal
27	N	N	N	N	low	low	N	N	N	N	Motor axonal
28	N	N	N	N	N	low	N	N	N	N	Motor axonal
29	N	N	N	N	N	low	N	N	N	N	Motor axonal
30	N	N	N	N	N	low	N	N	N	N	Motor axonal
31	N	low	N	N	low	low	N	N	N	N	Mixed
32	N	low	N	N	N	N	N	N	N	N	Pure sensory axonal
33	N	N	N	N	N	low	N	N	N	N	Motor axonal
34	N	N	N	N	N	low	N	N	N	N	Motor axonal
35	N	N	N	N	low	low	N	N	N	N	Motor axonal
36	N	N	N	N	low	low	N	N	N	N	Motor axonal
37	N	N	N	N	low	low	N	N	N	N	Motor axonal
38	N	N	N	N	N	low	N	N	N	N	Motor axonal
39	N	N	N	N	N	low	N	N	N	N	Motor axonal
40	N	N	N	N	low	low	N	N	N	N	Motor axonal
41	N	N	N	N	low	low	N	N	N	N	Motor axonal
42	N	N	N	N	N	low	N	N	N	N	Motor axonal

When the missed doses due to neuropathy were considered, 35 cases with available data were evaluated. The diagnoses of these patients are shown in Table 3. The mean amount of missed doses of vincristine in all these cases, except cases with leukemias, was 13.6 mg/m². While the mean amount of missed doses of vincristine in leukemias was 9 mg/m², it was found that only one case had a relapse and the other cases were in complete remission. While the mean missed dose was 24.9 mg/m² (37.7% of the total dose of vincristine according to their protocol) in patients with low-grade glioma, one patient was followed up with relapse, one case with progressive disease, and eight cases with stable condition. In the case of rhabdomyosarcoma with progression, the missed dose was 22.5 mg/m² (46.8% of the total dose of vincristine according to his protocol). While the mean dose missed in six medulloblastoma cases was 6.5 mg/m² (30.9% of the total dose of vincristine for the standard risk group according to their protocol, 24% for the high-risk group), four patients were in follow-up for complete remission, one for relapsed disease (standard risk), and the other for progressive disease (high risk). In two non-Hodgkin lymphoma cases observed in complete remission, the missed dose was 2.25 mg/m² (18.75% of the total dose of vincristine for the patient with T-cell NHL, 30% for the patient with B-cell NHL according to their protocol).

**Table 3:** Missed vincristine doses of respective diseases

Disease	n (%)	Missed vincristine dose (mg/m²)	Relapse/progressive disease rate (%)
Acute leukemia	15 (42.8)	9.00	6.60
Low-grade glioma	10 (28.5)	24.9	20.0
Medulloblastoma	6 (17.1)	6.50	33.3
Non-Hodgkin lymphoma	2 (5.88)	2.25	0.00
Rhabdomyosarcoma	1 (2.85)	22.5	100.00

Due to the insufficient number of patients, the cases were analyzed during the statistical analysis by grouping them into two groups: brain tumors (low-grade glioma and medulloblastoma) and hematological malignancies (acute leukemia and non-Hodgkin lymphoma). According to this analysis, no statistically significant difference was found between the diagnosis and the cumulative dose of vincristine or the duration of neuropathy (p=0.627, p=0.162). However, it was found that statistically significantly higher doses of vincristine were missed in the brain tumors group (p=0.031). When the missed vincristine dose was compared with the disease states, the missed dose of vincristine was lower in cases in complete remission compared to other cases. While no statistical difference was found in the analysis of cases with complete remission and cases with relapsed disease (p=0.4), a higher dose of vincristine was missed in the stable disease group than in the remission group (p=0.03).

## Discussion

Our study describes VIPN in children with malignancy. We showed that neuropathy developed in 7% of the 598 pediatric patients with vincristine treatment and that most of the cases were male. In literature, the frequency of severe VIPN in childhood is reported to be approximately 10% [1]. Although vincristine has poor oral bioavailability and poor blood-brain barrier penetration, it is an important chemotherapeutic agent used in many childhood cancers by taking advantage of its antimitotic effect [8]. Neuropathy is one of the most acute dose-limiting side effects of vincristine [9]. In a study by Pal et al., a motor abnormality was found in 18.7% of the cases receiving vincristine [10]. It is stated in the studies that there is no significant relationship between gender and VIPN [11]. In our study, most of the cases were male. There was no statistically significant relationship between gender and cumulative dose, and it was also found that gender did not affect the duration of neuropathy.

The median cumulative dose in patients with neuropathy was 17.23 mg/m², and although there was no statistically significant difference, the mean cumulative dose in patients with diffuse nerve involvement was found to be higher in our study. While it has been stated that severe VIPN is usually seen when a cumulative dose of 15-20 mg is reached [12], it is stated that neurotoxicity begins to be seen at cumulative doses of 6-8 mg [13]. Another study showed that neuropathy develops in 60% of the cases at cumulative doses of 30-50 mg of vincristine [14]. The lack of statistical difference in our research was thought to be related to the insufficient number of patients. The mean age of 42 patients analyzed for vincristine neuropathy in our study was 7.73±4.94 years. When the cases were investigated by grouping according to the median age of 7 years, it was observed that neuropathy developed with the cumulative dose of vincristine at statistically significantly lower doses in cases younger than seven. In the literature, it has been shown that the prevalence of VIPN generally increases at older ages [15], and a few studies have shown that the prevalence is higher in young children [16,17]. While neuropathy is expected more frequently in young children due to the immature neurological system and myelination, it is argued that it is less common in older children due to the faster vincristine clearance and the lower drug concentration in older children with the maximum vincristine dose of 2 mg [18-20]. In our study, neuropathy developed in patients younger than seven years of age, statistically significantly, at lower cumulative doses. Although the number of cases is small, our study contributes to the field of study showing that the younger age group may be a risk factor for the development of neuropathy, with this difference being statistically significant.

In most cases of VIPN, neuropathy progresses from distal to proximal; thus, the first presentation usually starts from the feet [2]. The most common symptoms are low feet and weakness in the extremities [21]. In our study, since the most common lower extremity was pure motor nerve involvement, the most common complaint was foot drop. As a result of the autonomic nervous system being affected, constipation, urinary retention, and orthostatic hypotension can be observed [22,23]. In our study, secondary globe vesicles were detected in only one case of urinary retention. Regarding constipation, constipation is occasionally seen in patients receiving chemotherapy and is controlled with laxative drugs. It is not easy to say that this finding is due to neuropathy caused by vincristine since it is thought that changes in diet and defecation habits during hospitalization with chemotherapy are also effective, apart from treatment. Therefore, these cases were not included in the study. Ptosis, one of the cranial neuropathies, is seen less frequently. In the series of our study, ptosis developed in three cases, and the findings were wholly regressed in three cases with vincristine discontinuation and pyridoxine treatment. In the literature, there are cases of vincristine-induced ptosis that completely regressed with pyridoxine [24,25], which is in line with our study.

The literature states that the findings may continue for months, depending on the severity of neuropathy [26]. In a study by Loprinzi et al., recovery from chemotherapy-induced neuropathy is reported to take approximately three months [21]. In a study in which cases with acute leukemia were evaluated, it was found that neuropathic symptoms persisted in 30% of the cases seven years after the cessation of treatment [27]. Another study showed that most patients who developed neuropathy recovered, and symptoms persisted in only 14% [28]. In a study in which a more detailed electrophysiological evaluation was performed in a similar patient group, electrophysiological neuropathy findings were shown in 33.75% of the cases [3]. In our study, the mean duration of neuropathy was 8.5 months, and sequelae were not detected in any of the cases. However, the fact that neuropathic improvement was only evaluated clinically in our study and nerve conduction study was not performed in every case because it was an invasive procedure is one of the factors limiting our study.

Studies have reported that VIPN is often dose-dependent [3,29], leading to the inability to take the required dose of vincristine [30]. One of the few studies about the effect of missed vincristine doses on cancer treatment success, evaluating neuropathy in low-grade glioma cases receiving carboplatin and vincristine treatment, showed that missed vincristine doses adversely affected the course of the disease [28]. They attributed this result, which is not compatible with the literature, to a large number of cases with BRAFV600E mutation, which adversely affected the prognosis, and to the fact that most dose reductions were made during the induction period. In our study, it was found that the missed dose of vincristine was lower in the cases in complete remission compared to the other cases. The fact that no statistical difference was found in the analysis of cases with complete remission and cases with a relapsed disease was attributed to the small size of the patient group. In the stable disease group, skipping a higher dose of vincristine compared to the remission group, which was statistically significant, also showed the importance of the missed vincristine doses in the treatment.

## Conclusions

Peripheral neuropathy due to vincristine remains an important side effect in childhood cancer treatment, especially affecting the lower extremities and sensory-motor involvement. Findings are usually reversible, thanks to early diagnosis and discontinuation of vincristine therapy. It can be encountered in less cumulative doses, mainly in the younger age group. Missed doses of vincristine may affect treatment success, and more comprehensive studies are needed to show this effect more clearly.
